# Measuring Food Insecurity in Children under 5 Years of Age with Acute Undernutrition in Valle Del Cauca—Colombia

**DOI:** 10.3390/children11101155

**Published:** 2024-09-24

**Authors:** Laura Valentina Parra-Pinzon, Elisa Maria Pinzon-Gomez, Sayda Milena Pico-Fonseca, Isabel Cristina Hurtado, Ana Rocio Guzman-Benavides, Olmer Alexander Pantoja-Rodríguez

**Affiliations:** 1 Institución Universitaria Escuela Nacional del Deporte, Santiago de Cali 760042, Colombia; 2 Facultad de Ciencias de la Salud, Universidad San Martin, Cali 111711, Colombia; 3 Secretaria de Salud Departamental, Gobernación Valle del Cauca, Cali 760045, Colombiaana.rocio.guzman@correounivalle.edu.co (A.R.G.-B.); 4 Ciencias de la Salud, Pontificia Universidad Javeriana Seccional Cali, Cali 760021, Colombia; 5 Department of Pediatrics, Universidad del Valle, Cali 760001, Colombia

**Keywords:** malnutrition in children under 5 years, food insecurity, ELCSA scale

## Abstract

Malnutrition is a global problem that affects all countries in one or more of its forms, representing one of the greatest challenges worldwide. One of the key contributing factors is food insecurity, which must be evaluated in children with moderate and severe acute malnutrition, as they are at imminent risk of death. Objective: Our objective was to assess food insecurity among children under 5 years old with moderate, and severe malnutrition from Valle del Cauca, a state located in the southwestern region of Colombia. Methods: A descriptive observational study was conducted, including children whose weight-for-height (W/H) indicator was below 2 SD, as recorded on the World Health Organization (WHO) growth charts, or who exhibited severe malnutrition phenotypes such as marasmus or Kwashiorkor. Family and child food security were evaluated using the Latin American and Caribbean Food Security Scale (ELCSA). Results: 58.6% of households with acutely malnourished children experienced food insecurity. A statistically significant relationship was found between food insecurity and children of Afro-Colombian and Indigenous descent. According to caregivers’ perceptions, 30.2% of child malnutrition cases were related to poor feeding and caregiving practices. Conclusions: Not all children with acute malnutrition suffer from food insecurity. Therefore, the findings of this research suggest that governmental efforts should focus not only on ensuring food availability, but also on educating caregivers about the importance of a balanced and nutritious diet tailored to the specific characteristics of each region and promoting appropriate caregiving practices.

## 1. Introduction

Malnutrition is a global issue affecting all countries in various forms, making it one of the most significant challenges worldwide. Malnutrition encompasses undernutrition, characterized by a caloric deficit, as well as overweight and obesity, which result from excessive caloric intake [1]. Children are among the most vulnerable groups to malnutrition, with approximately 45% of deaths among children under five years of age globally attributed to undernutrition [2]. In 2022, the United Nations (UN) and the World Food Programme reported that approximately 30 million children in the countries most affected by the current food crisis suffer from acute malnutrition, with 8 million experiencing severe acute malnutrition, the most lethal form of the condition [3].

In Colombia, the incidence of malnutrition among children under five years of age increased by 18.9% in 2022, with 20,336 cases reported. In Valle del Cauca, the third-largest department in the country, 432 cases of acute malnutrition were documented, corresponding to a prevalence of 0.30% per 100 children under five years of age [4,5].

Malnutrition in early childhood is particularly detrimental, as it can lead to brain disorders, including delays in overall development, motor skills, memory, and cognitive abilities. Furthermore, malnutrition can cause immunosuppression, increasing the risk of developing non-communicable diseases. At a population level, a malnourished child population imposes a significant economic burden on countries, as these individuals may not achieve full physical and mental development, leading to increased healthcare costs and a strain on the economically active population [6,7,8,9]. Malnutrition is driven by multiple causal factors, with food insecurity being a critical determinant [10]. The Food and Agriculture Organization of the United Nations (FAO) defines food insecurity as “the limited or uncertain availability of nutritionally adequate and safe food, or the limited and uncertain ability to acquire adequate food in socially acceptable ways” [11]. According to the 2022 Regional Panorama of Food and Nutrition Security, the number of people experiencing moderate to severe food insecurity in Latin America and the Caribbean increased from 205.2 million in 2019 to 267.7 million in 2021 [12].

Food and nutrition security is a fundamental human right, defined as the condition “when all people, at all times, have physical, social, and economic access to food that is safe and consumed in sufficient quantity and quality to meet their dietary needs and food preferences for a healthy and active life” [13]. Household food insecurity is a significant problem with wide-ranging impacts on child development, including reduced productivity; impaired mental, cognitive, behavioral, socio-emotional, and physical performance; and increased risks of mental health problems and chronic diseases [14,15,16].

The COVID-19 pandemic exacerbated food insecurity, leading to a global increase in hunger. World hunger rose in 2021 after remaining relatively stable since 2015. The prevalence of undernourishment increased from 8.0% to 9.3% between 2019 and 2020 and continued to rise at a slower pace in 2021, reaching 9.8% [17].

Although food insecurity has been extensively studied, its specific impact on children with acute malnutrition remains inadequately understood. This knowledge gap is crucial, as understanding access to and consumption of food within this population is essential for guiding immediate actions. To address this gap, the objective of this study is to assess food insecurity among children under 5 years old with moderate and severe malnutrition from Valle del Cauca, a state located in the southwestern region of Colombia. Addressing these factors will facilitate the development of targeted strategies, programs, and public policies aimed at reducing recidivism and mortality, ultimately improving the health outcomes of this vulnerable population.

## 2. Materials and Methods

A descriptive, cross-sectional observational study was conducted targeting children with acute, moderate, and severe malnutrition in Valle del Cauca, a department in southwestern Colombia comprising 40 municipalities and two districts, with an approximate population (excluding districts) of 2 million inhabitants, of which 7.2% are under five years of age. The study included children reported to the national epidemiological surveillance system (SIVIGILA) in 2022 whose weight-for-height indicator was below −2 standard deviations according to the World Health Organization (WHO) growth charts or who presented with severe malnutrition phenotypes such as marasmus or Kwashiorkor.

Probability sampling was carried out to estimate the prevalence of food insecurity. The sampling frame considered was children with malnutrition reported to the surveillance system during 2021, which corresponded to 340 minors. With an expected proportion of food insecurity of 50%, a confidence level of 95%, and a design effect of 1%, we obtained 70 patients in total, representing 20.6% of the reference population. For sample selection, all minors reported from epidemiological week 26 to week 40 of 2022 were considered.

One week after a case of acute malnutrition was reported, telephone contact was made with the caregiver. During this call, family and child food security were assessed using the Latin American and Caribbean Food Security Scale (ELCSA), a 14-question instrument that evaluates dimensions such as food quality, quantity, hunger, and concerns about access to food (Appendix A). The ELCSA was selected due to its low cost, rapid application, and validation in Colombia, as well as its use in national nutritional status surveys (ENSIN) [10].

Data were electronically recorded using Microsoft Excel 2013. For statistical analysis, the statistical program R version R 4.3.1 was used. Quantitative variables were reported as means or medians, with dispersion measures such as standard deviation and interquartile range depending on the normality of the data distribution. Categorical variables were described using absolute values and percentages. Frequency tables were created based on the presence or absence of food insecurity. Each of the variables was compared with the Student *t* test or Wilcoxon Mann–Whitney test for quantitative variables and the Chi square or Fisher exact test (when the expected values in any of the cells of the contingency table were less than 5) for categorical variables, according to compliance with assumptions. This study was approved by the research and ethics committee of the Escuela Nacional del Deporte and the research committee of the Departmental Secretariat of Health of Valle del Cauca.

## 3. Results

Seventy children reported to SIVIGILA were evaluated; 48.5% were between 1 and 2 years old, 52.8% were female, 14.2% identified as Afro-Colombian or Indigenous, 2.8% were foreigners, and 88.5% came from low socioeconomic backgrounds. In Colombia, socioeconomic stratification classifies residential properties primarily to apply differential rates for public utilities. The low and medium socioeconomic levels (1, 2, and 3) include households with the fewest resources, which are eligible for public service subsidies [18].

Regarding family characteristics, 21.3% of the children’s mothers reported having higher education, 47% of households had more than four residents, and 28.5% of these households included another child under five years of age (Table 1).

In terms of clinical characteristics, 75.7% of the children presented with moderate acute malnutrition, 17% had concurrent chronic malnutrition, 4.2% exhibited edema, 20% were born before 37 weeks of gestation, and 28.9% had adequate birth weight (Table 1). At the time of malnutrition diagnosis, 6 out of 10 children were underweight, and approximately 3% had hair abnormalities.

All children reported having been breastfed, with a median exclusive breastfeeding duration of 4 months and a median complementary breastfeeding duration of 9 months (Table 1).

**Figure 1:**
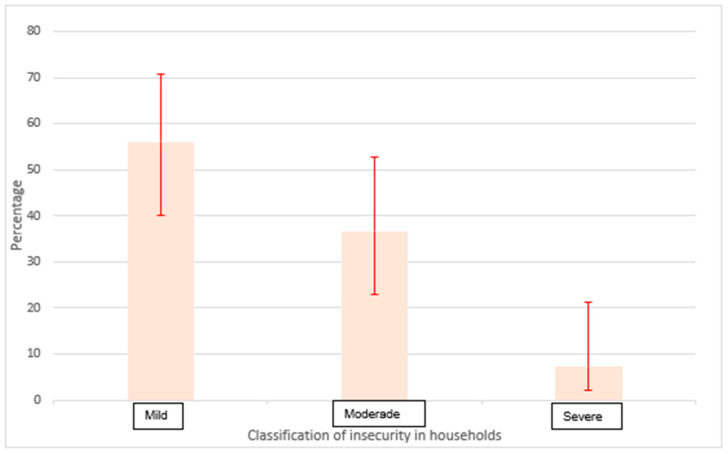
Classification of insecurity in households with the presence of a child under 5 years of age with acute malnutrition.

Food insecurity was identified in 58.6% of households, with 56% of these cases classified as mild (Figure 1). The ELCSA scale indicated that the primary factor contributing to food insecurity was concern about food running out, followed by concerns regarding food quality. When analyzing the ELCSA scale stratified by age groups (over and under 18 years), it is evident that within households facing difficulties in meeting basic needs, children are the least affected. Specifically, when there is a need to stop eating, reduce portion sizes, decrease the number of meals per day, or limit food variety, caregivers tend to prioritize the diets of younger children over those of individuals older than 18 years. This coping strategy reflects a protective approach towards children during food scarcity. Notably, 48% of households with children experiencing acute malnutrition did not report food insecurity, and in families with food insecurity, hunger-related dimensions showed low prevalence (Table 2).

To complement this analysis, the perceived causes of malnutrition were examined according to caregivers. In 30.2% of cases, malnutrition was attributed to inadequate feeding and parenting practices. Specifically, parents mentioned that the child refuses to eat due to laziness to chew, a preference for ultra-processed foods, or other reasons. Additionally, 25% of caregivers attributed malnutrition to genetic factors, while 14.4% were unsure of the cause. Less common factors included pre-existing diseases, cultural beliefs, or disbelief in the diagnosis. An association between malnutrition and food scarcity was reported by a minority of caregivers, consistent with the low prevalence of hunger-related dimensions on the ELCSA scale, which did not exceed 4%.

A bivariate analysis was conducted to identify variables for inclusion in the multivariate analysis. With the exception of ethnicity, the chi-square test did not indicate a significant relationship between food insecurity and the sociodemographic and clinical variables in this study (Table 3).

## 4. Discussion

In the present study, the prevalence of food insecurity was 58.6%. Similar studies worldwide have reported food insecurity rates ranging from 11% to 100%, depending on the population and its vulnerability [5,7,19]. In Colombia, approximately 54.2% of the population reported a perception of inadequate access to food, with estimates ranging between 50% and 65% [20]. An exploratory review of studies published from 1990 to July 2021 found that food insecurity remains a persistent problem, with a prevalence close to 12%.

In high-income countries, food insecurity rates are significantly higher in disadvantaged communities. For instance, in the United States, 35.3% of households with incomes below the poverty level experience food insecurity, while in Australia, up to 25% of households in low-income areas are affected [14,21].

It might be assumed that all children with acute malnutrition suffer from food insecurity. Indeed, studies have linked food insecurity with stunted growth, showing that the likelihood of growth retardation in children is higher in food-insecure households, sometimes up to 23 times greater than in food-secure households [8,22,23].

While numerous studies have examined the relationship between household food insecurity and children’s nutritional status, the results have been inconsistent. Some studies reported a positive association, while others found no relationship or even a negative association [24]. The present research shows that more than half have shown an association between malnutrition and food security, but 41.4% involved malnourished children who did not show food insecurity, which exposes the need to explore other factors related to malnutrition in children under 5 years of age in low-income countries.

Analysis of the dimensions of the ELCSA scale revealed that concerns about food running out, followed by issues related to the quality of food, were the most frequent. This aligns with coping strategies reported in other studies, which indicate that one of the main strategies to combat food insecurity is the consumption of lower-quality foods [20,23]. Substituting higher-quality foods with cheaper, less nutritious, and calorie-dense alternatives can result in inadequate nutrient and calorie intake, adversely affecting a child’s health. It is well known that suboptimal intake of energy, proteins, and micronutrients in the first five years of life can limit neuronal plasticity and impair cognitive functioning [21].

The ELCSA scale includes questions at both the household and child levels [25]. A stratified analysis showed that children are less exposed to food insecurity compared to other household members, reflecting a shift from traditional practices where the household head historically received the best food in terms of quality and quantity. These findings underscore the need for further research into the factors influencing this phenomenon.

When investigating factors related to food insecurity, it was found that ethnic background (specifically Indigenous or Afro-Colombian children) increases the likelihood of experiencing food insecurity. This finding is consistent with other studies and may be explained by the precarious environmental and biological conditions that affect the quality of life in these communities, which are often associated with poverty and are determinants of chronic malnutrition [26,27].

Contrary to expectations, this research found that not all children with acute malnutrition suffer from food insecurity, with the proportion of children experiencing hunger being very low. This suggests the need to explore other causal factors of malnutrition. National public health strategies should therefore consider approaches beyond complementary feeding programs and social assistance, taking into account the diverse factors that contribute to malnutrition.

To effectively target social programs, it is essential to evaluate food access and quality in terms of diversity and to assess the specific needs of different populations to ensure they benefit from these programs. New strategies for the management and recovery of children with malnutrition will undoubtedly be necessary.

The findings are consistent with the causes reported by the guardians of malnourished children, where lack of food was cited in a very low proportion of cases. Instead, factors such as inadequate parenting and feeding practices were more prominent. This highlights the need for educational interventions that address parenting practices and feeding guidelines, challenging myths and cultural beliefs. Health professionals play a crucial role in educating parents or guardians, making it imperative that academic programs ensure the acquisition of these skills.

Interestingly, 100% of mothers reported breastfeeding their children, with a median duration of exclusive breastfeeding of 4 months—higher than the national median reported in Colombia. This could be linked to the association between low income and low socioeconomic status, which may act as a protective factor for breastfeeding due to the economic barriers to accessing formula milk. Alternatively, an informant bias may be present, as malnutrition is often associated with neglect or abandonment, leading to an overestimation of breastfeeding practices. Further studies are needed to explore this association.

Finally, a significant proportion of children under 5 years of age who were malnourished were not food insecure. This finding indicates that, while access to food may not be a problem, other factors are contributing to childhood malnutrition. One possible explanation is the emergence of new parenting trends, particularly those related to permissiveness in infant feeding. These practices allow children to freely choose what to eat, which, although promoting autonomy and independence, can lead to unbalanced food choices lacking in essential nutrients.

In many cases, children opt for foods high in sugar and fat but low in protein, vitamins, and minerals. This unbalanced diet can result in malnutrition despite the availability of food. Parents influenced by these new parenting trends may be less likely to impose restrictions or guide their children’s food choices toward more nutritious options. Additionally, it is crucial to consider that parents’ perceptions of healthy eating may be distorted by advertising and marketing of food products that are portrayed as “healthy” but do not provide the necessary nutrients for proper child development.

Among the strengths of this research is its contribution to generating data on a relatively unexplored topic, as well as the use of validated scales such as the ELCSA. Among the limitations is that the data are part of a routine surveillance system and, therefore, were not collected for research purposes and may present difficulties in quality and coverage, which are minimized through interviews with caregivers one week after the case is reported to the epidemiological surveillance system. These findings pave the way for addressing malnutrition from the perspective of parenting practices, focusing on appropriate feeding techniques from the start of complementary feeding, exposure to diverse food textures, knowledge of healthy eating guidelines, and respectful parenting in relation to the child’s diet within the context of the family’s dietary habits.

## 5. Conclusions

The findings of this research underscore the importance of not only ensuring the availability of food, but also educating parents on the significance of a balanced and nutritious diet. It is essential to promote parenting practices that integrate child autonomy with appropriate nutritional guidance, thereby ensuring that children’s food choices support their health and development. To address this issue effectively, it is recommended to implement comprehensive nutritional education programs targeting both parents and caregivers. Additionally, policies should be developed to regulate the marketing of foods directed at children, ensuring that the promotion of healthy and balanced nutrition begins at an early age.

Therefore, based on the variables examined in this study, which are those monitored by the surveillance system, it is not possible to fully explain food insecurity. It is recommended that the Colombian surveillance system include information that includes other biological and social factors that allow for a better understanding and explanation of the situation of malnutrition in children under 5 years of age. Further research is justified on other possible biological and social factors in malnutrition in children under 5 years of age.

## Figures and Tables

**Table 1:** Sociodemographic and clinical characteristics of children aged 0 to 59 months in Valle del Cauca—Colombia.

Variable		*n*	%	IC 95%
Age	0 to 11 months	10	14.2	(7.74–24.87)
	12 to 35 months	34	48.5	(36.87–60.42)
	36 to 59 months	26	37.1	(26.44–49.27)
Sex	Male	33	47.1	(35.53–59.06)
	Female	37	52.8	(40.93–64.46)
Ethnicity	Ethnic	10	14.3	(7.7–24.87)
	No ethnic	60	85.7	(75.12–92.25)
Socioeconomic level *	Low	62	88.6	(78.46–94.28)
	Medium	8	11.4	(5.71–21.53)
Nationality	Colombia	68	97.1	(88.94–99.3)
	Foreign	2	2.9	(0.69–11.05)
Mother’s educational level	Primary	25	35.7	(25.18–47.83)
	Secondary	30	42.9	(31.58–54.92)
	Superior	15	21.4	(13.18–32.87)
Number of people in the household	Less than four	37	52.9	(40.93–64.46)
	Four or more	33	47.1	(35.53–59.06)
Presence of other children under 5 years old	Yes	20	28.6	(19.03–40.5)
	No	50	71.4	(59.49–80.96)
Degree of acute malnutrition	Moderate (W/H −2 to −2.99 SD)	53	75.7	(64.03–84.51)
	Severe (W/H < 3 SD)	17	24.3	(15.48–35.96)
Chronic malnutrition	Delay in size	12	17.1	(9.86–28.12)
	Risk of short stature	17	24.3	(15.48–35.96)
	Right size	41	58.6	(46.47–69.71)
Edema	Yes	3	4.3	(1.34–12.78)
	No	67	95.7	(87.21–98.65)
Prematurity	Yes	14	20.0	(12.06–31.3)
	No	56	80.0	(68.69–87.93)
Birth weight	Underweight (<2499 g)	20	28.6	(19.03–40.5)
	Efficient weight (2500–2999 g)	22	31.4	(21.45–43.46)
	Appropriate weight (≥3000)	28	40.0	(28.99–52–11)
Visible thinness **	Yes	42	60.0	(47.88–71)
	No	28	40.0	(28.99–52.11)
Changes in hair	Yes	5	7.1	(2.93–16.34)
	No	65	92.9	(83.65–97.06)
Breastfed minors		100%		
Median exclusive breastfeeding		4 months		
Median complementary breastfeeding		9 months		

Source: SIVIGILA–Own survey. * In Colombia, the socioeconomic level of families is established based on access to financial, educational, social, and health resources; therefore, those who are classified as low are those who have less access to these services. ** Clinical assessment described by the WHO attached in the epidemiological surveillance form.

**Table 2:** ELCSA scale dimensions by age group.

Questions	Dimension	% of Minors under 18 Years of Age Who Experienced It	% of Adults Who Experienced It
In the last 3 months, due to lack of money or other resources, have you stopped eating healthy?	Quality and quantity of food	28.5	38.5
In the last 3 months, due to lack of money or other resources, have you had a diet based on little variety of foods?	Food quality	28.5	40
In the last 3 months, due to lack of money or other resources, have you stopped eating breakfast, lunch or dinner?	Amount of food	8.5	21.4
In the last 3 months, due to lack of money or other resources, did you eat less than you should have?	Amount of food	8.5	30
In the last 3 months, due to lack of money or other resources, have you felt hungry but not eaten?	Hunger	4.2	14.2
In the past 3 months, due to lack of money or other resources, did you only eat once a day or did you skip eating for a whole day?	Hunger	4.2	8.5
In the last 3 months, due to lack of money or other resources, have you ever had to reduce the amount served at meals?	Quantity and quality of food	4.2	

**Table 3:** Sociodemographic characteristics associated with food insecurity in children with acute malnutrition.

Sociodemographic Factors		Food Insecurity				*p* Value Chi Square or Fisher’s Exact
		Yes		No		
		*n*	%	*n*	%	
Age	0 to 11 months	6	14.63	4	13.79	0.516 *
	12 to 35 months	22	53.66	12	41.38	
	36 to 59 months	13	31.71	13	44.83	
Sex	Male	16	39.02	17	58.62	0.106 **
	Female	25	60.98	12	41.38	
Ethnicity	Ethnic	9	21.95	1	3.45	0.038 *
	Non-ethnic	32	78.05	28	96.55	
Socioeconomic level	Low	39	95.12	23	79.31	0.059 *
	Medium	2	4.88	6	20.69	
Nationality	Colombia	40	97.56	28	96.55	1 *
	Foreign	1	2.44	1	3.45	
Mother’s educational level	Primary	16	39.02	9	31.03	0.257 **
	Secondary	19	46.34	11	37.93	
	Superior	6	14.63	9	31.03	
Number of people in the household *	Less than four	21	51.22	16	55.17	0.744 **
	Four or more	20	48.78	13	44.83	
Presence of other children under 5 years old	Yes	12	29.27	8	27.59	0.878 **
	No	29	70.73	21	72.41	
Degree of acute malnutrition	Moderate	29	70.73	24	82.76	0.248 **
	Severe	12	29.27	5	17.24	
Chronic malnutrition	Delay in size	20	48.78	9	31.03	0.285 **
	Right size	21	51.22	20	68.97	
Edema	Yes	3	7.32	0	0	0.261 *
	No	38	92.68	29	100	
Prematurity	Yes	7	17.07	7	24.14	0.467 **
	No	34	82.93	22	75.86	
Birth weight	Low weight (<2499 g)	13	31.71	7	24.14	0.774 **
	Efficient weight (2500 g−2999 g)	16	39.02	12	41.38	
	Adequate weight (≥3000)	12	29.27	10	34.48	
Visible thinness	Yes	23	34.48	19	65.52	0.428 **
	No	18	43.9	10	34.48	
Changes in hair	Yes	5	12.2	0	0	0.07 *
	No	36	87.8	29	100	

Source: SIVIGILA–Own survey. * Fischer’s test was applied. ** Chi square applied.

## Data Availability

The data presented in this study are available upon request from the corresponding author due to privacy.

## Appendix A. Latin American and Caribbean Food Security Scale (ELCSA)

The following table presents the 14 questions included in the Latin American and Caribbean Food Security Scale (ELCSA) which are used to assess food security in households:

**Table:**

Question	Wording
1	In the last three months, was there ever a time when you or any household member ran out of food due to lack of money or other resources?
2	In the last three months, was there ever a time when you or any household member felt worried that food would run out before you could buy more?
3	In the last three months, was there ever a time when you or any household member ate less balanced meals due to lack of money or other resources?
4	In the last three months, was there ever a time when you or any household member ate less than they should because of lack of money or other resources?
5	In the last three months, was there ever a time when you or any household member had to reduce the size of meals or skip meals because of lack of money or other resources?
6	In the last three months, was there ever a time when you or any household member went a whole day without eating due to lack of money or other resources?
7	In the last three months, was there ever a time when you or any household member ate less because you were worried that the food would run out?
8	In the last three months, was there ever a time when you or any household member ate less nutritious food because of lack of money or other resources?
9	In the last three months, was there ever a time when you or any household member had to borrow food or money to buy food?
10	In the last three months, was there ever a time when you or any household member had to ask for food from neighbors, relatives, or friends due to lack of money or other resources?
11	In the last three months, was there ever a time when you or any household member had to cut down on the quantity of food consumed because of lack of money or other resources?
12	In the last three months, was there ever a time when you or any household member had to skip meals for a whole day due to lack of money or other resources?
13	In the last three months, was there ever a time when you or any household member had to rely on cheaper but less nutritious food due to lack of money or other resources?
14	In the last three months, was there ever a time when a child in the household went without food for an entire day due to lack of money or other resources?

Note: Each question must be answered with “Yes” or “No”. Affirmative responses indicate different levels of food insecurity. Source: FAO. Latin American and Caribbean Food Security Scale (ELCSA)-Manual for Use and Application [Internet]. 2012. 9p. Available from: https://www.fao.org/3/i3065s/i3065s.pdf, accessed on 2 September 2024.
